# Prevalence of mental distress among adult survivors of childhood cancer in Germany—Compared to the general population

**DOI:** 10.1002/cam4.1936

**Published:** 2019-03-06

**Authors:** Juliane Burghardt, Eva Klein, Elmar Brähler, Mareike Ernst, Astrid Schneider, Susan Eckerle, Marie Astrid Neu, Arthur Wingerter, Nicole Henninger, Marina Panova‐Noeva, Jürgen Prochaska, Philipp Wild, Manfred Beutel, Jörg Faber

**Affiliations:** ^1^ University Medical Center Mainz Mainz Germany

**Keywords:** anxiety, common mental disorders, depression, long‐term survival, pediatric cancer, psychological distress

## Abstract

**Background:**

Increasing survival rates after childhood cancer have raised the issue of long‐term mental health consequences in adulthood. This study determines mental health distress among long‐term survivors of pediatric cancer and compares it to control groups.

**Methods:**

Childhood cancer survivors (CCS; N = 951, aged 24‐49 years) were compared to three age‐matched control groups from the general population collected at three time points. The study compared the prevalence of clinically relevant symptoms of a wide range of common mental disorders (depression, somatic distress, suicidal ideation, generalized anxiety, panic, social anxiety, and sleep disturbances) using identical, validated questionnaires. CCS were identified by the German Childhood Cancer Registry. Controls were approached by a demographic consultation company (USUMA) which assured that the three samples were nationally representative.

**Results:**

Childhood cancer survivors reported higher education than controls and were less often married. All forms of common mental distress were increased among survivors. Twenty‐four percent of male (N = 526) and 41% of female survivors (N = 425) reported some form of clinically relevant mental health symptoms. Somatic distress as the leading complaint was highly frequent among CCS (OR: 10.98, CI 95%: 7.24‐16.64). Complaints by generalized anxiety (OR: 5.04, CI 95%: 2.61‐9.70), panic (OR: 3.28, CI 95%: 1.60‐6.70), depression (OR: 3.36, CI 95%: 2.22‐5.09), and suicidality (OR = 2.22; CI 95%: 1.38‐3.57) were also strongly increased. Female sex, low education, low income, and unemployment were associated with increased distress.

**Conclusions:**

Findings indicate a need to integrate psycho‐oncological screening and care into long‐term aftercare. Somatic distress, as cause and indicator of psychological distress, should receive stronger attention, especially tiredness, low energy, and pain.

## 1 INTRODUCTION

The increasing proportion of children surviving cancer has promoted a strong interest in somatic and psychological late consequences. Stressful and traumatic experiences of cancer and its treatment^1^, ^2^, ^3^, ^4^ may compound developmental challenges of childhood and enhance the risk to suffer from distress. A review of studies of childhood brain tumor survivors^5^ concluded that survivors were at risk to develop a broad range of mental disorders (ie, depression, anxiety, suicidal ideation, behavioral problems). Survivors within the American Childhood Cancer Survivor Study (CCSS) reported more mental distress than their siblings^6^: The study found increased scores of anxiety, depression, somatization, and combined, comorbid scores among adult survivors of childhood cancer using profiles of the Brief Symptom Inventory (BSI). Similarly, survivors of childhood leukemia, Hodgkin's disease, and non‐Hodgkin's lymphoma had an increased risk of depression and somatic distress.^7^ A register‐based German study reported elevated risks of clinically relevant symptoms of posttraumatic stress, anxiety, and/or depression among long‐term survivors of cancer in adolescence compared to healthy controls (peers, recruited by leaflets, and email snowball‐system).^2^ Additionally, Dieluweit, Debatin, Grabow, Kaatsch, Peter, Seitz, and Goldbeck^8^ reported indicators for delayed social development (more men living on their own, fewer women with marriage and parenthood) among survivors.

On the other hand, a Swiss cohort study found less than average psychological distress in adult survivors of childhood cancer compared to the norm of the BSI.^9^ However, the proportion of survivors with an increased Global Severity Index was comparably large; their distress scores were in the range of psychotherapy outpatients.

Regarding determinants of distress, studies have concurred that female sex was associated with higher distress.^2^, ^9^ Additional risk factors were being a single child, age >30 years, and immigrant status.^9^

Many previous findings were based on American cohorts, notably the CCSS.^10^ National differences may influence mental health burden, for instance, the lack of mandatory health insurance in the United States. The absence of an insurance was associated with increased mental distress among survivors.^11^ Studies have focused on depression or global measures of mental distress^11^, ^12^ while data on other symptoms such as anxiety is scarce.^13^ In contrast, this study measures a wide range of common mental disorders, especially generalized anxiety, panic, social anxiety, sleep disturbances, suicidal ideation, in addition to depression. Among them is somatic distress, which refers to any burden by somatic symptoms irrespective of whether they were caused by somatic changes following the cancer treatment or mental processes. They are important factors influencing perceived quality of life.^14^

Many analyses have compared mental distress among CCS to siblings.^5^, ^6^ While ensuring comparability regarding genetic endowment and environment, siblings may also suffer from increased stress in the patients’ families.^7^, ^15^

We therefore compared a large cohort of long‐term (≥23 years since diagnosis) adult survivors of childhood cancer (CCS) with age‐matched samples from the general population. The aims of this study were to (a) comprehensively assess the prevalence of distress by common mental disorders among CCS, (b) to compare the prevalence to the general population, and (c) to identify associations of distress to sociodemographic characteristics.

## 2 PATIENTS AND METHODS

### 2.1 Procedure

#### 2.1.1 CCS sample

Data were collected in the Cardiac and vascular late sequelae in long‐term survivors of childhood cancer (CVSS‐) study^16^ and analyzed as part of the add‐on study PSYNA (Psychosocial long‐term effects, health behavior, and prevention among long‐term survivors of cancer in childhood and adolescence). The CVSS‐study identified survivors using the nationwide German Childhood Cancer Registry (GCCR). This registry systematically documents patients with childhood cancer residing in Germany since 1980 and is largely exhaustive.^17^ German CCS were included in the CVSS‐study, who were diagnosed with neoplasia according to the International Classification of Childhood Cancer (ICCC 3) between 1980 and 1990 while they were 0‐15 years old. They were registered at the GCCR, survived more than 5 years since diagnosis, and received cancer treatment at one of the 34 participating cancer centers, which were in acceptable travel distance from the study center in Mainz, Germany. In agreement with the German Association of Childhood Oncology survivors of Hodgkin lymphoma and nephroblastoma were excluded. Of 2894 eligible survivors who were invited, 1002 participated in the examination between September 2013 and February 2016; 51 survivors were excluded due to subsequent malignant neoplasm (ICCC3); resulting in 951 participants. At the study center, participants completed a standardized 5.5‐hour examination including cardiovascular and clinical phenotyping, self‐administered questionnaires, and a computer‐assisted personal interview.^18^

#### 2.1.2 Comparison samples

Three representative face‐to‐face household surveys of the German population (age range 14‐95 years) were conducted in 2006, 2008, and 2010 by the demographic consultation company USUMA (Berlin) using identical procedures.^19^, ^20^, ^21^ Participants answered questionnaires on their own, in the presence of a professional interviewer. All participants provided verbal informed consent. To match the age range of the CVSS‐cohort, only participants aged 20‐49 years served as comparison groups.

In the 2006 survey, a total of 1287 individuals (61.9% of all valid addresses)^19^ reported their symptoms of social phobia,^22^ generalized anxiety,^23^ and panic.^24^ In the age‐group of 20‐49 years, 569 participants (57.1% female) were analyzed. Somatic distress,^25^ depression,^26^ and suicidality^27^ were assessed in 2008. Of 2524 participants (62.8% of all eligible),^21^ 1130 participants (52.8% female) aged 20‐49 years were included. Sleep disorders^28^ were measured in 2010 in a total sample of 2520 participants (61.9% of all eligible),^20^ resulting in a comparison group of 1054 individuals (54.1% female) aged 20‐49 years.

### 2.2 Study measures (CVSS and controls)

The Patient Health Questionnaire (PHQ‐8)^29^ quantifies the frequency of being bothered by eight diagnostic criteria of major depression over the past 2 weeks. As in the other PHQ scales and items below, ratings ranged from 0=“not at all,” 1=“several days,” 2=“over half the days,” to 3=“nearly every day.” Sum scores ≥10 indicated a case of depression; previously yielding a sensitivity and specificity of 88% for major depression.^26^ Suicidal ideation was assessed by the item “In the last 2 weeks, have you been bothered by thoughts that you would be better off dead, or of hurting yourself?”. Caseness was defined by answers ≥1 (“several days”).^27^ Generalized anxiety was measured with the GAD‐2.^23^ Sum scores ≥3 indicated generalized anxiety with good sensitivity (86%) and specificity (83%).^30^ Panic disorder was identified using affirmative responses to the first two items of the brief PHQ panic module.^24^ Social anxiety was assessed by the three items of the German versions of the Mini‐Social Phobia Inventory (Mini‐Spin).^22^, ^31^ Caseness was identified by sum scores ≥6.^31^ The PHQ‐15 asked subjects to rate the severity of 15 symptoms over the last 4 weeks. Sum scores ≥10 were used to identify increased symptom burden.^25^ Sleep disorders were identified using the Jenkins sleep scale by scores ≥3, that is, problems with sleep on an average of at least one night a week.^28^

In the CVSS, demographic variables were measured within the computer‐assisted personal interview, while controls answered them as part of the questionnaire. The variables included marital status, living in a partnership, education, and unemployment (Table 1). Education was recoded into the categories: Low (≤9 years, “Hauptschule” or no graduation”), middle (10 years, “Realschule”), and high (≥12 years, “Abitur”) to prevent small category sizes. Monthly household income was measured using 24 categories (eg, from 0€‐150 € to >20 000€). We analyzed household income using the median of the category borders. Values above 20 000 were equated to 20 000.

**Table 1:** Demographical characteristics of female and male survivors of childhood cancer (CVSS)‐study participants

	CVSS total N = 951	CVSS women N = 425 44.7%	CVSS men N = 526 55.3%
Age, median (range)	34.2 (24‐49)	33.6 (24‐48)	34.6 (24‐49)
Household income in €, median (range)^a^	2875.0 (75‐20 000)	2625.0 (75‐20 000)	3125.0 (75‐20 000)
Years since diagnosis, median (range)	28.1 (23‐36)	28.1 (23‐36)	28.0 (23‐35)
Age at diagnosis, median (range)	5.0 (0‐15)	4.4 (0‐15)	5.7 (0‐15)
Age‐group, % (N)			
20‐29 y	23.9 (227)	28.0 (119)	20.5 (108)
30‐39 y	58.4 (555)	56.5 (240)	59.9 (315)
40‐49 y	17.8 (169)	15.5 (66)	19.6 (103)
Age at diagnosis, % (N)			
<1 y	9.5 (90)	10.8 (46)	8.4 (44)
1‐<4 y	30.8 (293)	33.2 (141)	28.9 (152)
4‐<8 y	27.7 (263)	28.7 (122)	26.8 (141)
8‐<11 y	13.5 (128)	11.8 (50)	14.8 (78)
11‐<15 y	18.6 (177)	15.5 (66)	21.1 (111)
Diagnosis			
Leukemias	43.5 (414)	45.6 (194)	41.8 (220)
Lymphomas	9.9 (94)	6.4 (27)	12.7 (67)
CNS tumors	12.8 (122)	13.2 (56)	12.5 (66)
Neuroblastoma	7.6 (72)	8.2 (35)	7.0 (37)
Retinoblastoma	1.1 (10)	0.7 (3)	1.3 (7)
Renal tumors	8.1 (77)	10.8 (46)	5.9 (31)
Hepatic tumors	0.7 (7)	0.7 (3)	0.8 (4)
Bone tumors	5.3 (50)	4.2 (18)	6.1 (32)
Soft tissue sarcoma	7.5 (71)	6.4 (27)	8.4 (44)
Germ cell tumors	2.7 (26)	3.3 (14)	2.3 (12)
Carcinoma	0.7 (7)	0.5 (2)	1.0 (5)
Others	0.1 (1)	0.0 (0)	0.2 (1)
Marital status, % (N)^b^			
Married	37.2 (354)	36.2 (154)	38.0 (200)
Never married	58.0 (552)	56.5 (240)	59.3 (312)
Divorced	4.0 (38)	5.6 (24)	2.7 (14)
Separated	0.6 (6)	1.4 (6)	0.0 (0)
Cohabiting with partner, yes, % (N)^c^	57.0 (540)	57.5 (244)	56.5 (296)
Unemployed, yes, % (N)^d^	3.2 (27)	4.0 (14)	2.6 (13)
Educational level, % (N)^e^			
Low (≤9 y, “Hauptschule”/no graduation/“other")	14.0 (133)	11.1 (47)	16.5 (87)
Moderate (10 y, “Realschule”)	27.0 (256)	33.1 (140)	22.0 (116)
High (≥12 y, “Abitur”)	59.0 (560)	55.8 (236)	61.5 (324)

Missing values:

a household income: women 54/women 36,

b marital status: women 1/men 0,

c cohabiting with partner: women 1/men 2,

d unemployed: women 79/men 32,

e educational level: women 2/men 0.

### 2.3 Statistical analysis

Descriptive measures were reported as absolute numbers/percentages, means with 95% confidence intervals or median, and range for demographic variables and mental distress. To compare the prevalence of mental distress between CVSS participants and the general population, we conducted binary logistic regressions on the dichotomized self‐report measures of mental distress (depression, somatic distress, suicidal ideation, generalized anxiety, panic, social anxiety, and sleep disturbances) as dependent variables. Each regression model included the independent variables group membership (CVSS vs control), sex, age, marital status, education, income, and unemployment. To analyze factors influencing mental distress, we conducted binary logistic regressions with the same factors excluding group membership. Somatic distress was analyzed with a multivariate analysis of variance with the 15 items as dependent variables, age as covariate, and the factors sex and group (CVSS vs control). All *P*‐values correspond to two‐tailed tests. As this is an exploratory analysis, *P*‐values are reported without correction for multiple testing. Therefore, *P*‐values should be treated with caution and are given for descriptive reasons only. Statistical analyses were performed using SPSS 23 (IBM, Armonk, NY, USA) for Windows.

## 3 RESULTS

### 3.1 Demographics

Table 1 presents demographic and medical characteristics of the CCS participants, separately for men and women. Fifty‐five percent were male and 45% were female. Median age was 34 (range 24‐49) years. Men reported higher income than women. The median age at cancer diagnosis was 5 years, 28 years prior to the survey. Prevailing diagnoses were leukemia, CNS tumor, lymphoma, renal, neuroblastoma, soft tissue, and bone tumor.

Table S1 compares CCS participants to the sociodemographic characteristics of the different controls. Control samples were in a comparable age range; however, their median ages were slightly higher (37‐38 vs 34 years). Median household income was higher among survivors. Considerably, fewer survivors were married (37%) compared to the control samples (48%‐54%). More survivors had never married (58% vs 34%‐37%); correspondingly, their divorce rates were lower. However, survivors and controls did not differ regarding cohabitation with a partner (survivors: 57% controls: 59%‐65%). Education was higher among survivors with 59% reporting a high, 27% a middle, and 14% a low degree. The control samples reported lower levels of education with only 18%‐19% holding a high, 44%‐45% a middle, and 36%‐37% a low degree. The rate of unemployment was lower among CCS (3%) vs controls (9%‐10%).

### 3.2 Mental distress

**Figure 1:**
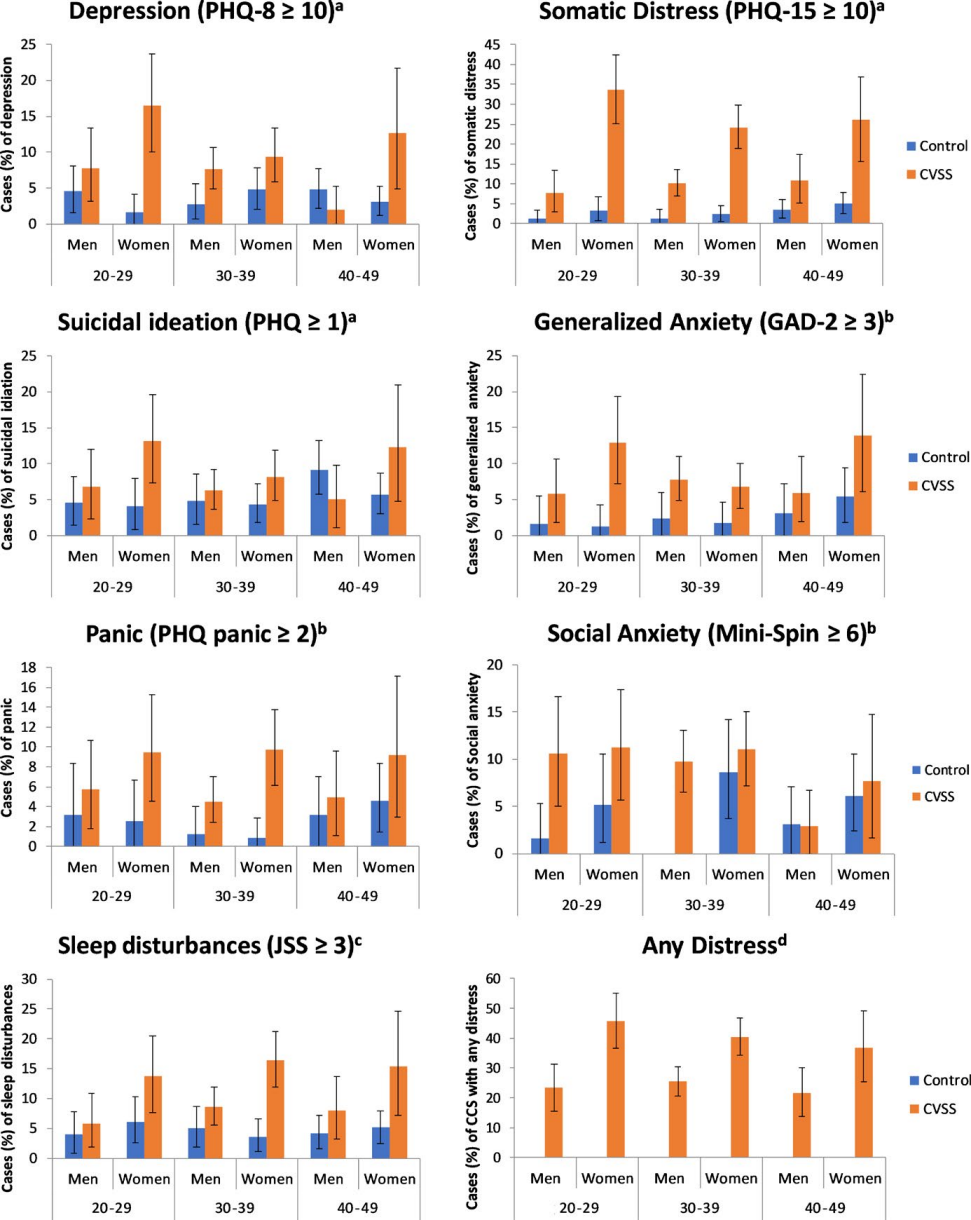
Presented are percentages over the respective cut‐offs of each scale and CI 95% among men and women from the general population (compared to ^a^control group 2008, ^b^control group 2006; no cases of social anxiety among men aged 30‐39 y; ^c^control group 2010) and survivors of childhood cancer (CVSS); ^d^“Any distress”: occurrence of at least one of the mental conditions (depression, somatic distress, suicidal ideation, generalized anxiety, panic, social anxiety, or sleep disturbances) “Any distress” was only available for the CVSS sample

Figure 1 shows the prevalence of specific forms of mental distress among survivors for men and women per age‐group (Table S2 displays the prevalence per sex combining all age‐groups.). Descriptively, the most prevalent complaints reported by CCS were increased somatic distress (18%), sleep disturbances (11%), social anxiety (9%), and depression (9%). Among controls, social anxiety (5%), sleep disturbances (5%), and depression (4%) were the leading symptoms, followed by somatic distress (3%). CCS reported suicidal ideation and generalized anxiety in 8% of cases (controls: 6% and 3%; respectively), and panic in 7% of cases (controls: 3%). Overall, 32% of survivors reported some form of clinically relevant distress (“any distress”); these were 24% of male and 41% of female survivors.

Regarding sex, the most frequent complaints among male CCS were somatic distress 10% (controls: 2%), social anxiety 9% (controls: 2%), and sleep disturbances 8% (controls: 4%). Further, 7% of male CCS reported depression (controls: 5%) and generalized anxiety (controls: 2%), each. Six percent reported suicidal ideation (controls: 7%) and 5% panic (controls: 2%).

Female CCS suffered most frequently from somatic distress (27%; controls: 4%), sleep disturbances (15%; controls: 5%), and depression (12%; controls: 3%). Further, 11% of female CSS suffered from social anxiety (controls: 7%), in 10% of cases from panic (controls: 3%), generalized anxiety (controls: 3%), and suicidal ideation (controls: 5%) each.

Table 2 shows the results of the logistic regressions comparing the CCS‐cohort to the control samples, accounting for demographical factors and the logistic regressions predicting mental distress among CCS survivors from said demographical factors. All forms of mental distress were increased among long‐term survivors, in particular (*P *< 0.001) somatic distress, generalized anxiety, panic, depression, suicidal ideation, and sleep disturbances. Among CCS, women suffered more frequently from high somatic distress (*P *= 0.00001), panic (*P *= 0.026), and sleep disturbances (*P *= 0.030) compared to men. Higher age was associated with decreased depression (*P *= 0.004). Being married was associated with a lower risk for suicidal ideation (*P* = 0.014). Lower education was associated with increased depression, somatic distress, and generalized anxiety: Compared to high education, low education increased the chance to be burdened by depression (*P* = 0.031), somatic distress (*P* = 0.002), and generalized anxiety (*P* = 0.011). Compared to high education, medium education increased the chance to be burdened by somatic distress (*P* = 0.0001) and generalized anxiety (*P *= 0.008). Higher income was associated with a lower risk to be burdened by somatic distress (*P* = 0.005). Being unemployed was associated with increased risk to suffer from generalized anxiety (*P *= 0.003) and panic (*P* = 0.029)

**Table 2:** Mental distress (depression, somatic distress, suicidal ideation, generalized anxiety, panic, social anxiety, and sleep disorders) in CCS vs controls: Impact of sociodemographic variables

	Depression^a^	Somatic distress^a^	Suicidal ideation^a^	Generalized anxiety^b^	Panic^b^	Social anxiety^b^	Sleep disorders^c^
Group (controls = 0, CVSS=1)^d^	**4.69 [2.70, 8.16]**	**20.89 [11.68, 37.36]**	**2.22 [1.38, 3.57]**	**7.66 [3.41, 17.22]**	**4.36 [1.88, 10.09]**	**2.75 [1.40, 5.42]**	**2.32 [1.14, 4.73]**
Sex (male=0, female=1)^e^	1.23 [0.69, 2.18]	**2.56 [1.68, 3.91]**	1.15 [0.65, 2.03]	1.11 [0.63, 1.96]	**1.97 [1.09, 3.58]**	1.17 [0.70, 1.95]	**1.73 [1.05, 2.84]**
Age^e^	**0.92 [0.87, 0.97]**	0.96 [0.92, 1.00]	0.99 [0.94, 1.04]	0.96 [0.91, 1.01]	0.97 [0.91, 1.02]	0.98 [0.93, 1.03]	1.01 [0.96, 1.05]
Marital status (not married=0, married=1)^e^	1.21 [0.62, 2.36]	1.24 [0.76, 2.01]	**0.37 [0.17, 0.82]**	0.73 [0.37, 1.43]	1.33 [0.67, 2.64]	0.58 [0.31, 1.07]	1.01 [0.58, 1.77]
Education^e^							
Low (=1) vs high (=0)	**2.33 [1.08, 5.02]**	**2.57 [1.40, 4.69]**	1.41 [0.61, 3.26]	**2.68 [1.25, 5.73]**	1.58 [0.71, 3.53]	1.34 [0.63, 2.85]	1.62 [0.78, 3.33]
Middle (=1) vs high (0)	1.84 [0.97, 3.48]	**2.57 [1.63, 4.07]**	1.72 [0.92, 3.23]	**2.36 [1.25, 4.45]**	0.85 [0.42, 1.72]	1.32 [0.74, 2.37]	1.56 [0.90, 2.70]
Income in 100€ per month^e^	0.98 [0.96, 1.00]	**0.98 [0.96, 0.99]**	0.99 [0.97, 1.01]	0.99 [0.98, 1.01]	0.98 [0.96, 1.01]	1.00 [0.99, 1.01]	0.99 [0.98, 1.01]
Unemployed (employed = 0, unemployed = 1)^e^	2.63 [0.88, 7.88]	1.54 [0.57, 4.19]	3.26 [1.17, 9.14]	**4.57 [1.68, 12.37]**	**3.39 [1.14, 10.10]**	1.35 [0.38, 4.79]	0.90 [0.25, 3.24]

Bold print *P* < 0.05 two‐tailed.

a Compared to control group 2008.

b Compared to control group 2006.

c Compared to control group 2010.

d Logistic regression models (Odd ratios and CI_95%_) including survivors of childhood cancer (CVSS) vs controls group, sex, age, marital status, education, income in 100€, employment.

e Logistic regression models (Odd ratios and CI_95%_) among survivors of childhood cancer (CVSS) including sex, age, marital status, education, income in 100€, employment.

Based on the PHQ‐15, CCS participants reported consistently more symptoms of somatic distress than controls (*F*(1, 1896) = 515.83, *P < *0.000001, $\eta_{p}^{2}$ = 0.21). Overall, women suffered more frequently from somatic distress than men (*F*(1, 1896) = 126.66, *P < *0.000001, $\eta_{p}^{2}$ = 0.06). This sex difference was bigger among CVSS participants (*F*(1, 1896)=28.91, *P <* 0.000001, $\eta_{p}^{2}$ = 0.02) than in the general population, though the size of this interaction was small. Age was statistically significant, however the effect size too small to be meaningfully interpreted (*F*(1, 1896) = 6.95, *P* = 0.008, $\eta_{p}^{2}$ = 0.004). The four most frequently reported symptoms were feeling tired/low energy, trouble sleeping, headaches, and back pain among both men and women.

## 4 DISCUSSION

Findings from a large, register‐based sample of 526 men and 425 women highlight the long‐term mental health burden among adult survivors of childhood cancer; on average 28 years after diagnosis: Common mental distress affected a total of 32% of the CCS sample (41% of women, 24% of men). Participants most frequently reported increased somatic distress (18%), sleep disturbances (11%), and social anxiety (9%). Among somatic distress, CCS were especially burdened by feeling tired/low energy, trouble sleeping, headaches, and back pain. Women suffered more frequently from mental distress than men: Increased somatic distress (27% vs 10%), sleep disturbances (16% vs 8%), and panic (5% vs 3%). These sex differences are in line with findings from the general population.^32^, ^33^ Regarding aspects of somatic distress, differences between female and male survivors slightly exceeded those in the general population.

Adult CCS reported common mental distress (depression, somatic distress, suicidal ideation, GAD, panic, social anxiety, and sleep disorders) more frequently than age‐matched samples from the general population. The strongest differences were found for somatic distress, generalized anxiety, panic, and depression when sex, age, marital status, education, income, and unemployment were controlled.

Our study complements previous findings regarding the prevalence of overall burden by mental health complaints. Earlier work had demonstrated that 14% of survivors of cancer in adolescence were affected by posttraumatic stress, depression, or anxiety.^2^ Screening for symptom profiles the American CCSS found 38% of survivors suffering from elevated mental distress,^6^ which is comparable to our study where 32% of survivors reported significant mental health symptoms. Single symptoms were also comparably frequent as in other reports. For instance, the high prevalence of suicidality of 8% corresponded to the CCSS.^34^ A similar prevalence was also reported for somatic distress and depression.^11^ However, American survivors reported higher rates of sleep disturbances (CCSS: 17% vs CVSS: 11%). The CCSS did not include measures for panic or social anxiety. Comparing the CVSS to a smaller French cohort instead showed no increased risk for panic and social anxiety among French survivors.^35^ Overall, the comparison of the German and American CCS cohorts shows similar results despite cultural and socioeconomic differences (eg, Germany's mandatory health insurance). However, these findings should be compared with caution as they are based on different self‐report measures.

The findings on disturbed sleep and low energy are in line with the notion that fatigue is a frequent complaint among survivors.^36^, ^37^, ^38^ A recent review concluded that chronic severe pain was a relevant long‐term effect of cancer among 5%‐10% of survivors.^39^

Survivors of childhood cancer participants were less often married than controls, which earlier American, British, and German studies considered a long‐term adverse psychosocial consequence of childhood cancer.^2^, ^40^, ^41^ On the other hand, participants of the CVSS sample were well educated compared to the representative samples. This mirrors findings from adolescent survivors in Germany,^2^, ^8^ while British CCS cohorts reported educational levels similar to controls, except for specific tumors.^42^ Regarding determinants of distress, unemployment as well as lower education and income were associated with increased distress. The protective effect of high social status may explain why the majority in our CCS sample (68%) was unburdened by any of the assessed mental disorders.

### 4.1 Strengths and limitations

The large, register‐based sample was screened for common mental disorders, covering a uniquely broad range of mental distress by standardized self‐report measures. Survivors have been recruited in early to middle adulthood, when adverse medical consequences such as heart failure have begun to manifest as subclinical syndromes. The CVSS data were compared to three individual control groups from the general population. While these control groups were sampled following identical procedures, they were recruited in three years (2006‐2010). While the unemployment rate among CVSS participants was relatively low for entire Germany (where the controls were recruited), it was in line with the comparatively low rates of the study region. Alternatively, the high level of education may have reduced unemployment.

Two control groups (control 2010 and control 2008) were not screened for earlier cancer diseases. Thus, it is likely that some cancer survivors were included within the representative samples. However, data from the third control group showed that only 2 individuals (0.4%) of the sample reported having suffered from cancer. Thus, these individuals should only have a small effect on the results.

The response rate among invited cancer survivors (32.9%) was acceptable taking into consideration that CVSS participants had to travel to the study center and comparable to similar studies (eg, 43.5%^2^ where questionnaires were sent home). However, a bias by self‐selection must be considered: Healthy individuals, without any long‐term effects, might identify themselves less strongly with their disease and therefore might drop out. On the other hand, individuals with high somatic, cognitive, or psychological burden may have declined participation because questions regarding their former disease might cause strong distress. Thus, the effect of selective dropout is difficult to evaluate. Yet, preliminary analysis of study participants and invited nonparticipants showed that these two groups were very similar with respect to sex, age at diagnosis, year of diagnosis, and diagnosis.^43^

The high prevalence of mental distress reflects stresses sustained by cancer and its treatment in childhood and its long‐term consequences. While our data indicate good educational, social, and vocational adjustment, they point to considerable long‐term emotional problems. These may be reactivated by follow‐up examinations, resp. risks, and manifestations of long‐term somatic complications. Thus, our data indicate a need to integrate psycho‐oncological screening and care into long‐term aftercare considering sociodemographic risk factors.^44^ Screening should cover a broad range of common mental disorders. Attention should be paid to somatic distress, in particular fatigue and pain symptoms. Future analyses will determine mental health care use and needs in this population vulnerable both to somatic and psychological adverse long‐term consequences.

## CONFLICT OF INTEREST

We declare no conflicts of Interest.
