# Supplementary material for: Prevalence of mental distress among adult survivors of childhood cancer in Germany—Compared to the general population
Source: Cancer Med. 2019 Mar 6;8(4):1865–74. doi: 10.1002/cam4.1936 (PMC6488141; doi:10.1002/cam4.1936)
Supplement: Supplementary file 1 [file CAM4-8-1865-s001.docx]

**Suppl Table 1. Demographical characteristics of childhood cancer survivors (CVSS) and control groups**

|  | **CVSS**  ***N* = 951** | **Control 2006**  ***N* = 569** | **Control 2008**  ***N* = 1,130** | **Control 2010**  ***N* = 1,054** |
| --- | --- | --- | --- | --- |
| **Age, median (range)^a^** | 34.2 (24-49) | 37.0 (20 - 49) | 38.0 (20 - 49) | 38.0 (20 - 49) |
| **Household income in €, median**  **(range/ *N* missing)** | 2,875  (75 – 20,000/ 90) | 1,750 (250 – 5,000/ 17) | 2,250 (250 – 5,000/ 47) | 2,250 (250 – 5,000/ 25) |
| **Age group, % (*N*)^a^** |  |  |  |  |
| 20 – 29 yr | 23.9 (227) | 24.8 (141) | 24.7 (279) | 26.0 (274) |
| 30 – 39 yr | 58.4 (555) | 35.3 (201) | 31.6 (357) | 31.3 (330) |
| 40 – 49 yr | 17.8 (169) | 39.9 (227) | 43.7 (494) | 42.7 (450) |
| **Marital status, % (*N*)** |  |  |  |  |
| married | 37.2 (354) | 53.6 (305) | 52.1 (589) | 48.0 (506) |
| never married | 58.0 (552) | 34.8 (198) | 33.9 (383) | 36.9 (389) |
| divorced | 4.0 (38) | 9.8 (56) | 11.9 (134) | 12.7 (134) |
| widowed | 0 (0) | 0.5 (3) | 0.6 (7) | 0.8 (8) |
| separated | 0.6 (6) | 1.2 (7) | 1.5 (17) | 1.6 (17) |
| *N* missing | 1 | 0 | 0 | 0 |
| **Cohabiting with partner, yes, % (*N*)** | 57.0 (540) | 64.7 (368) | 62.1 (702) | 59.4 (626) |
| *N* missing | 3 | 0 | 0 | 0 |
| **Unemployed, yes, % (N)e** | 3.1 (84) | 8.1 (46) | 7.2 (81) | 9.3 (98) |
| *N* missing | 84 | 0 | 0 | 0 |
| **Education, % (*N*)** |  |  |  |  |
| low (9 years, “Hauptschule“) | 14.0 (133) | 36.7 (209) | 37.4 (423) | 36.1 (380) |
| moderate (10 years, “Realschule”) | 27.0 (256) | 45.3 (258) | 44.3 (501) | 45.2 (476) |
| high (12–13 years, “Abitur”) | 59.0 (560) | 17.9 (102) | 18.2 (206) | 18.8 (198) |
| *N* missing | 2 | 0 | 0 | 0 |

Representative control groups 2006: depression (PHQ8, suicidality), somatic distress (PHQ-15); REP 2008: generalized anxiety (GAD-2), social anxiety (Mini-SPIN); REP 2010: sleep disorders (Jenkins Sleep Scale)

**Suppl Table 2. Prevalence in % [CI_95%_] of mental distress among survivors and controls for men and women.**

| **Mental distress** | **CVSS**  **total** | **Control total** | **CVSS**  **men** | **Control men** | **CVSS women** | **Controls women** |
| --- | --- | --- | --- | --- | --- | --- |
| **Depression** (PHQ-8 ≥ 10)^a^ | 9 [7-11] | 4 [3-5] | 7 [4-9] | 5 [4-9] | 12 [9-15] | 3 [2-5] |
| **Somatic distress** (PHQ-15 ≥ 10)^a^ | 18 [15-20] | 3 [2-4] | 10 [7-12] | 2 [1-4] | 27 [23-32] | 4 [2-5] |
| **Suicidal ideation** (PHQ ≥ 1)^a^ | 8 [6-10] | 6 [4-7] | 6 [4-8] | 7 [5-9] | 10 [7-13] | 5 [3-7] |
| **GAD** (GAD-2 ≥ 3)^b^ | 8 [6-10] | 3 [2-4] | 7 [5-9] | 2 [1-5] | 10 [7-13] | 3 [1-5] |
| **Panic** (PHQ panic ≥ 2)^b^ | 7 [5-9] | 3 [1-4] | 5 [3-7] | 2 [1-5] | 10 [7-13] | 3 [1-5] |
| **Social anxiety** (Mini-Spin ≥ 6)^b^ | 9 [8-11] | 5 [3-6] | 9 [6-11] | 2 [0-3] | 11 [8-14] | 7 [4-10] |
| **Sleep disturbances** (JSS ≥ 3)^c^ | 11 [9-13] | 5 [3-6] | 8 [6-10] | 4 [3-6] | 15 [12-19] | 5 [3-7] |
| **Any distress^d^** | 32 [29-35] | -^e^ | 24 [21-28] | -^e^ | 41 [37-46] | -^e^ |

**Note.** Presented are percentages over the respective cut-offs of each scale and CI_95%_ among men and women from the general population (compared to ^a^ control group 2008, ^b^ control group 2006; ^c^ control group 2010) and survivors of childhood cancer (CVSS); ^d^ “Any distress”: occurrence of at least one of the mental conditions (depression, somatic distress, suicidal ideation, generalized anxiety, panic, social anxiety, or sleep disturbances); ^e^“Any distress” was only available for the CVSS sample.
